# Hot and Cool Executive Functions in Middle Childhood: Evidence for a Weak but Process-Specific Relationship

**DOI:** 10.3390/children13081109

**Published:** 2026-08-19

**Authors:** Eva Košíková, Daniela Turoňová, Ľubica Konrádová, Barbora Mesárošová

**Affiliations:** 1 Department of Psychology, Faculty of Arts, Comenius University, Gondova 2, 811 02 Bratislava, Slovakia; lubica.konradova@uniba.sk (Ľ.K.); barbora.mesarosova@uniba.sk (B.M.); 2 The Research Institute of Child Psychology and Pathopsychology, Cyprichova 42, 831 05 Bratislava, Slovakia; daniela.turonova@vudpap.sk

**Keywords:** executive functions, Hot, Cool, decision-making under uncertainty, cognitive flexibility, interaction, feedback learning, middle childhood, IGT, WCST

## Abstract

**Highlights:**

**What are the main findings?**

- Cool (WCST) and Hot (IGT) executive functions showed a weak and selective association in children.
- WCST post-loss reaction time predicted IGT learning, while global WCST indices did not.

**What are the implication of the main finding?**

- Adaptive decision-making relates more to feedback processing than to global cognitive flexibility.
- The findings support a process-based and developmentally sensitive view of executive functioning.

**Abstract:**

**Background:** Executive functions (EFs) are cognitive and emotional abilities that underpin goal-directed behavior, decision-making, and problem-solving. EFs can be categorized into two broad types, Hot EFs and Cool EFs, distinguished by their relation to emotional processing. Cool EFs are associated with cognitive control, while Hot EFs involve emotional regulation and decision-making in emotionally charged contexts. EFs contribute to self-regulation, including the ability to adjust behavior in response to feedback. **Methods:** The aim of this study is to investigate the relationship between Hot (decision-making under uncertainty) and Cool (cognitive flexibility) EFs in middle childhood. To assess these dimensions of EFs, we used two tasks, the Iowa Gambling Task (IGT) and the Wisconsin Card Sorting Test (WCST), completed by children (N = 90) aged 6–12 years. **Results:** Traditional WCST indices of cognitive flexibility were not associated with IGT performance. Using hierarchical linear regression models, the most significant overlap concerns the negative relationship between post-loss adjustment in the WCST (post-loss reaction time—plRT) and learning across the IGT (learning index—LI). Children who responded faster after negative feedback (plRT ↓) showed greater improvement in advantageous choice over the course of the task (LI ↑). **Conclusions:** The findings suggest that decision-making in the IGT is more closely linked to dynamic, process-based aspects of self-regulation than to static measures of cognitive flexibility. It can be concluded that the relationship between WCST and IGT during middle childhood is best described as weak, process-specific, and developmentally modulated, indicating that Cool and Hot EFs are partially distinct yet interconnected systems.

## 1. Introduction

Executive functions (EFs) are a set of higher-order cognitive processes involved in flexible, top-down regulation of behavior that supports goal-directed action [1,2,3]. These processes are essential for planning, monitoring, adapting, and controlling behavior in responses to changing demands [4]. EFs play a critical role in daily functioning across personal, work/academic, and social contexts [5,6]. They are not isolated functions, but are closely intertwined with other cognitive domains such as attention, memory, learning, and language [7,8]. Among several proposed structural models, the influential EF framework by Miyake et al. [2] identifies three core components: inhibition (of attention/response), shifting (between mental sets), and updating (of working memory). Other researchers have extended these components to reasoning, planning, problem solving, cognitive flexibility, and others [3,4,9]. Findings in the literature are often heterogeneous, and there is no clear consensus on which processes qualify as core EFs or how they should be conceptually defined [7,10,11]. Despite these theoretical limits, in developmental psychology, EFs are seen as foundational for self-regulation and adaptive functioning, and they are a central focus in both basic research and clinical assessment of neurocognitive development [12,13,14].

Another widely discussed aspect of EFs is the multidimensional nature of the construct, particularly the distinction between “Cool” EFs, which are primarily cognitive and abstract, and “Hot” EFs, which are engaged in situations involving emotion, motivation, or reward [15]. The Cool EFs are defined as emotionally neutral, strategic, coherent, and flexible, but often slower. Cool EFs typically include components described in Miyake’s model such as inhibition, updating, shifting, as well as components from other models such as planning, cognitive flexibility, etc. [9]. The Hot EFs are influenced by emotional processes and impulses, and therefore may operate more rapidly in emotionally or motivationally salient contexts. However, they are not necessarily “faster” in a general sense, but rather more strongly shaped by affective and reward-related processes. These include, for example, delay of gratification, risky decision-making, or affective learning [4]. Hot and Cool EFs are, in theory, distinct but correlated factors [16] and exist on a continuum [14]. The balance between these two systems is determined by the developmental level of the child, the child’s self-regulatory abilities, and experienced stress [17].

One practical reason for such division of EFs is the neurobiological basis of brain development and cognitive performance [18]. Age-related changes in cognitive performance reflect both quantitative and qualitative developmental shifts, which are closely related to the neurobiological maturation of frontal areas [9] and stabilization of neural networks in the brain [19]. Despite their importance, EFs remain a conceptually diverse group of processes, and there is still debate regarding their underlying structure—whether they function as a unified system or consist of separable components. One proposed solution to this is based on Miyake et al.’s [2] model of unity and diversity of EFs—EFs components are related to each other, but they not the same (critique of this model: Sambol et al., 2023 [20]). This model can be extended to account not only for the unity and diversity of components, but also for the dimensions of these components—namely, Hot and Cool EFs and their interrelations.

EFs also show evidence of integration or unity. Some reviews suggest that unity among EFs is more prominent in earlier childhood and then naturally gains heterogeneity [21]. For example, developmental research has demonstrated that Cool EFs components tend to operate in a more integrative manner in early childhood [22]. Because of these findings, some authors suggest that distinctions between individual Cool EFs become more pronounced with age and maturation. In younger children, differences in cognitive performance may reflect a general, undifferentiated executive capacity, whereas in older individuals, distinct EFs components such as inhibition, updating, and shifting become more clearly delineated [23].

Zelazo and Cunningham [8] proposed a model of emotion regulation grounded in both Hot and Cool EF principles, suggesting that these processes operate within a shared conceptual framework rather than as fully independent systems. Subsequent work supports this integrated view: Cool and Hot EFs components follow distinct but related developmental trajectories across middle childhood [4] and recruit common lateral prefrontal regions, despite being statistically dissociable [24].

Similarly, Hot and Cool EFs share a common neurobiological basis in general. Both rely on specific regions such as the dorsolateral prefrontal cortex (dlPFC), basal ganglia, and brainstem [25]. However, Hot EF processes are more strongly associated with intermediary brain areas that link cognitive and emotional processing [26]. For instance, cortex areas linked to specific Hot EFs are the anterior cingulate cortex, the orbitofrontal cortex, and the medial prefrontal cortex or insula. These regions are often viewed as functional mediators between the dlPFC and subcortical structures [27]. This distinction parallels broader neurocognitive models describing rapid subcortical emotional reactivity as distinct from, yet integrated with, cortically mediated cognitive–emotional regulation [28]. Together, these findings support the notion of unity–diversity patterns across different dimensions of EFs.

In this study, we focus on the diversity–unity model of Hot and Cool EFs. Specifically, we focus on cognitive flexibility as a Cool component and risky decision-making as a Hot component. In particular, we tested whether cognitive flexibility predicts variability in risky decision-making; that is, whether stronger Cool EFs are linked to more adaptive performance in hot, emotionally salient, or motivationally salient contexts.

Cognitive flexibility (CF) is commonly defined as the ability to adapt cognitive processes in response to changing demands of the world or shifting task rules [29]. It is often characterized by behavior requiring deployment of different strategies, reflecting dynamic changes in the environment [30]. Higher CF has been linked to more effective problem solving [31], better adaptation to novel or changing situations, higher creativity [32], etc. CF is often conflated with the shifting component of EFs, and shifting is often assessed using the same tasks as CF [29]. The main theoretical difference between them is that shifting is a more narrowly defined EF than CF. Some findings show that CF inherently contains other EFs, mainly inhibition and working memory [21]. This highlights one of the most common limitations of EFs studies: the validity of tests is lower than hypothesis-testing requires, and tests have often so called “impurities” [7,33].

We selected CF as the representative Cool EF because it is one of the most extensively studied EF components in both basic and clinical research and is theoretically meaningful for explaining individual differences in cognitive performance [34]. Although CF, like most EF measures, is subject to task impurity—reflecting contributions from working memory and inhibition—this is not a limitation for the present purposes: since our aim is to explore the unity–diversity relationship between Cool and Hot EFs rather than to isolate pure components, a task that inherently draws on multiple interacting EFs, it is well suited to this goal. Similarly, risky decision-making (Hot EF) is also assessed using a non-specific task involving multiple EFs. For CF, we use the Wisconsin Card Sorting Test (WCST).

To assess ambiguous and risky decision-making, we used the Iowa Gambling Task (IGT) [35]. This task is widely used to assess decision-making under uncertainty and emotional influence [36]. The task is based on choosing a card from decks, with subsequent reward and/or punishment, with the aim of winning as much money as possible. Herem the participant must figure out which card decks are risky and which are advantageous based on implicit feedback.

Both these tasks involve more than one EF, but both rely on implicit rules that must be discovered and successfully applied. The difference between the tasks is in the affective valence of feedback, which is motivational in the IGT but not in the WCST. Together, the WCST and IGT provide a complementary approach to investigating the unity and diversity of executive functioning in the context of flexibility in decision-making. The impurity of each task becomes a feature rather than a limitation, allowing us to explore how multiple EFs processes interact in a dynamic, multidimensional context.

While the WCST targets adaptation to abstract rule changes in a relatively neutral context, the IGT emphasizes regulation of behavior in emotionally salient, uncertain situations. By comparing performance across these two tasks, we aim to examine whether individual differences in Hot and Cool EFs performance reflect separate cognitive-emotional systems or whether they share common underlying mechanisms [37,38,39,40].

### Objectives

This study aims to test whether performance on the Wisconsin Card Sorting Test (WCST) statistically predicts performance on the Iowa Gambling Task (IGT)—that is, whether cognitive flexibility accounts for unique variance in risky decision-making. It is based on the idea that Hot and Cool EFs may represent partially distinct, yet related, aspects of executive control, particularly when considering their developmental trajectories, although prior findings have been mixed [41].

More specifically, we focus on the association between task performance assumed to index a Cool EF component (cognitive flexibility on the WCST) and a Hot EF component (risky decision-making on the IGT). Some studies have reported little or no clear relationship between such Hot and Cool EFs components, or between their task-based indicators [42,43], whereas other studies have reported associations in selected samples or for selected outcomes. For instance, Lehto and Elorinne [44] are often discussed in this context, but the exact WCST–IGT association (which indices, effect size, and statistical significance) is not reported precisely; therefore, we describe this evidence as mixed and potentially outcome-specific. Similarly, studies in adults [45] and clinical populations [46] have reported associations in some samples or metrics, but the results are heterogeneous and may depend on outcome definitions and population characteristics. The present study therefore approaches the WCST–IGT link as potentially outcome-specific rather than assuming a uniform association across all task metrics.

This study builds on the literature by testing whether WCST indices explain incremental variance in IGT performance after accounting for age. The goal is to contribute to the ongoing discussion about the differing developmental trends of Hot and Cool EFs. Specifically, this study hypothesizes that cognitive flexibility—a key factor in WCST performance—is also essential for success on the IGT, but we expect that any overlap may be modest once age is accounted for, consistent with mixed prior findings.

Given this multidimensionality of CF, the present study does not rely on a single global WCST score. Instead, it examines several theoretically relevant WCST indices in order to capture more specific cognitive mechanisms potentially linked to IGT performance. Particular attention is given to feedback-related adjustment in both tasks. In the WCST, feedback guides rule learning and set shifting; in the IGT, feedback informs subsequent choices under uncertainty and with a changing reward–loss experience. To maintain interpretability, feedback-related constructs were operationalized explicitly in both tasks.

**H1.**  *Better cognitive flexibility on the WCST (fewer perseverative errors (PEs), more conceptual level responses) will be associated with more adaptive decision-making on the IGT (higher net score).*

**H2.**  *More effective feedback-based adaptation on the WCST (fewer perseverative errors, shorter post-loss reaction time) will be associated with greater feedback-related learning across trials on the IGT (higher learning index).*

## 2. Methods

### 2.1. Research Sample

A total of 90 children who completed both tasks were included in the primary analyses; 47 were girls (M = 8.98 years, SD = 1.42), and 43 were boys (M = 8.58 years, SD = 1.53). The two groups did not differ significantly in age (t(88) = 1.28, *p* = 0.205).

Exclusion criteria were age other than 6.0–12.11 years; the presence of a neurodevelopmental (SLD, ASD), neurological, or psychiatric disorder or sensory and motor deficits (monitored through an anamnestic questionnaire); and the presence of an acute illness that could cause fatigue during testing.

The children’s fatigue was monitored using a nonverbal self-assessment Sleepiness Scale. Children who did not complete both the WCST and the IGT were excluded from the analyses. Additionally, a small number of participants were excluded from the specific model constructed using GORILLA Experiment Builder software© (v2022–v2024). Testing was conducted in a single session by trained administrators.

### 2.2. Research Tools, Outcome Variables, and Derived Indices

#### 2.2.1. Wisconsin Card Sorting Task (WCST-64)

The WCST is widely regarded as the gold standard for assessing executive functions, and its construct validity is well established [47,48]. However, the reliability evidence is mixed and index-dependent: a recent meta-analysis of test-retest coefficients found values generally ranging between 0.3 and 0.7 across indices [49], with earlier estimates as low as 0.46–0.64 for specific error scores [50], whereas split-half reliability has been reported to be considerably higher (≥0.90) for core indices in both a modified clinical version [47] and a self-administered computerized variant [51].

Cognitive flexibility was assessed using a computerized 64-card version of the Wisconsin Card Sorting Test [52], which is widely considered the gold standard for evaluating cognitive flexibility [47,48,53]. The WCST primarily assesses the ability to shift cognitive strategies in response to changing task demands and external feedback, independent of emotional influences [3].

**Figure 1:**
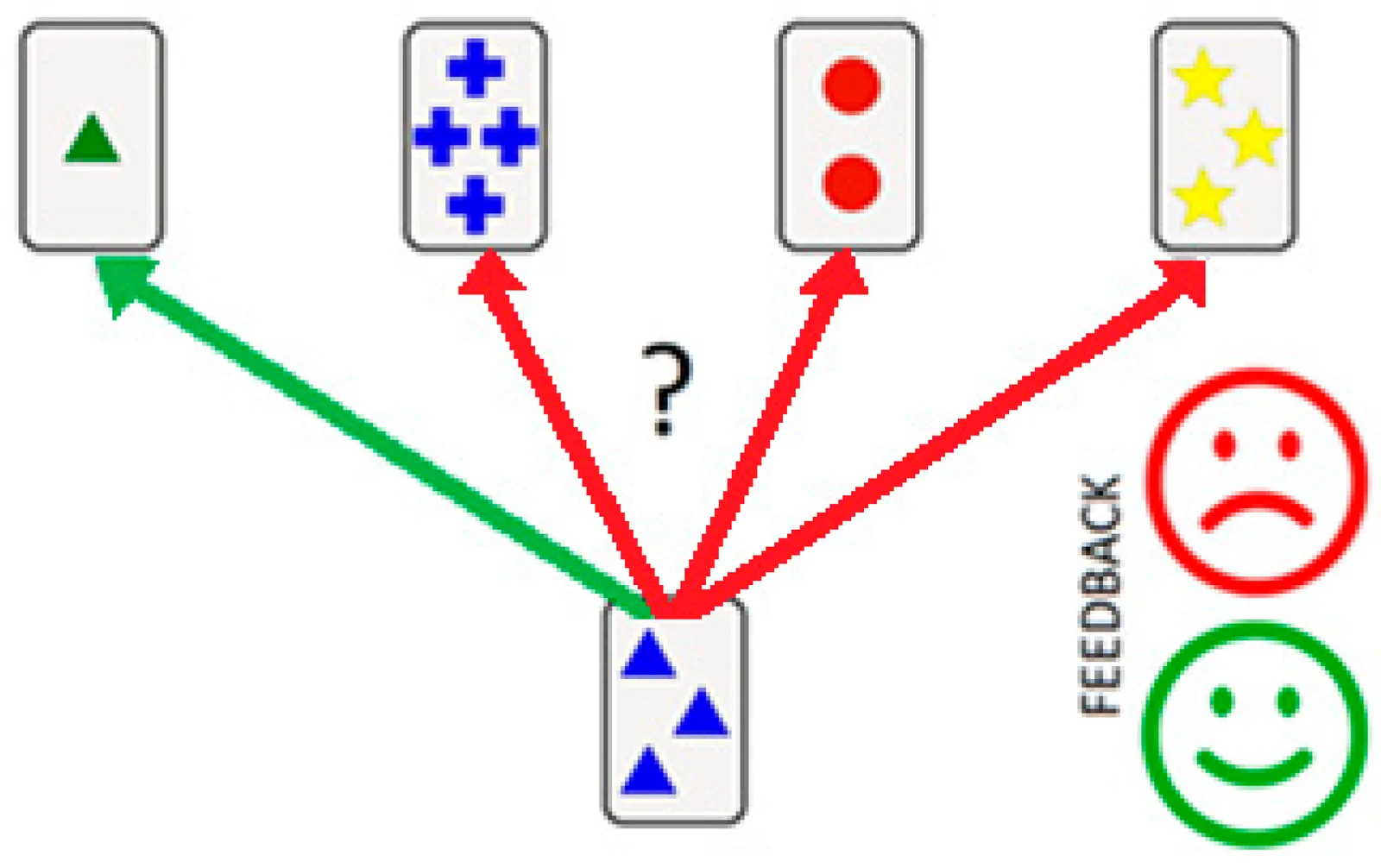
A trial example of the WCST, with the sorting rule “shape”.

In this task, participants are presented with four response cards and a target card. Each card contains a random number (1,2,3,4), a random shape (circle, triangle, cross, star), and a random color (red, green, blue, yellow). A sorting rule (number, shape, or color) unknown to the participants is in effect. The participants choose one of the response cards and get the feedback “correct” if the chosen response card matches the target card according to the sorting rule and the feedback “incorrect” otherwise (Figure 1).

The game consists of 64 trials in which the response cards and the target card change in every trial, so that at least one of the response cards matches the target card according to the sorting rule. The objective is to determine the unknown sorting rule based solely on trial-by-trial feedback and choose correct response cards in following trials. After 10 consecutive correct responses, the sorting rule in effect changes. The participants then have to repeat the process of determining the correct sorting rule and adjust the choice of the response cards to the new rule. Before the game begins, participants are informed that their objective is to choose the correct response cards according to a sorting rule that must be determined through trial and error. They are also informed that this rule may change over time and are asked to adjust their sorting strategy accordingly.

The test requires the use of working memory to maintain previous rules, inhibition to suppress interference from other stimulus dimensions, and cognitive flexibility to identify rule changes after negative feedback and adapt accordingly [54].

In our study, we used a custom-made PC version of the WCST, based on age-appropriate adaptations [55].

*WCST indices eligible for modeling (continuous predictors)*
  - Perseverative errors (PEs)—number of perseverative errors, i.e., situations when the participant continues with the previous (no longer valid) sorting strategy even after negative feedback. In our scoring, the first incorrect response following the old rule after a rule change was not yet counted as perseverative. Perseveration was counted only when the participant repeated the same outdated rule again after negative feedback, and each further response based on that old rule was counted separately, even if interrupted by correct answers.
  - PEs in the WCST primarily represent reactive behavioral flexibility. It is a critical index of feedback use. High values indicate a failure to adapt behavior following evidence of a rule change. Perseveration errors are a key indicator of cognitive flexibility: a large number of such errors indicates a problem with switching mental settings after a rule change.
  - Non-perseverative errors (NPEs)—number of errors that are not perseverative. NPE captures a broader range of mistakes, including random responding, misunderstanding of task rules, or attention lapses. NPE are all other errors in which the child’s response does not comply with the current rule, but unlike perseverative errors, they do not reflect repeated use of the previously valid rule. They may reflect unsystematic responding, rule confusion, or attentional lapses.
  - Conceptual level responses (CLRs)—number of responses that indicate the ability to maintain the correct sorting strategy (series of ≥3 correct responses in a row following the same sorting rule). In our scoring, if at least three consecutive correct responses occur in a set/category (that is a series of consecutive trials with the same sorting rule), the set/category contributes to CLR by 1. High CLR values indicate that feedback was used to form and maintain a correct rule concept, while low CLR suggests rule application without clear conceptualization or strategy.
  - Failure to maintain set (FMS)—the number of set-maintenance failures (i.e., occasions when the child breaks an established run of correct sorting before the rule changes). In our scoring, FMS was recorded only when the participant had already produced at least three consecutive correct responses and then made an error before completing the category (CC). Multiple consecutive errors within the same breakdown episode were counted as a single FMS event; several FMS events are possible during one set/category. Higher FMS is interpreted as inconsistent application of a known rule and may reflect lapses in sustained attention, transient loss of the current mental set, or distractibility (i.e., the child “knows” the rule but does not maintain it consistently). From a feedback perspective, FMS can be viewed as difficulty maintaining behavior in line with ongoing confirming feedback. Limitation: FMS is only informative once a sorting rule has been understood. If a child never reaches the criterion, there is no opportunity to observe set maintenance failures, and a low/zero FMS in that case does not imply good set maintenance; it may instead indicate a failure to understand the sorting rule. FMS was examined at the bivariate level but was not entered into regression models, as the primary focus of H1 was on indices directly reflecting flexible rule application rather than set maintenance failures.
*WCST Reaction Time Metric*

Post-loss reaction time (plRT) was calculated as the arithmetic mean reaction time on trials immediately following an incorrect WCST response (negative feedback), computed trial-by-trial (trial *t* was included if trial *t* − 1 was incorrect, including consecutive errors). Trials with reaction times below 200 ms were excluded as anticipatory responses, consistent with standard RT screening practice. Trials displaying repeated identical reaction time values were excluded as likely recording artifacts attributable to the Gorilla Experiment Builder platform; participants with missing RT data due to this criterion were excluded from plRT-based models (N = 4).

#### 2.2.2. The Iowa Gambling Task (IGT)

The IGT is a well-established paradigm for assessing decision-making under uncertainty, with reasonable support for its construct validity [56], although several validity and reliability concerns have also been raised [57]. Conventional summary scores have shown only moderate test–retest reliability (r = 0.27–0.36 depending on the retest interval; [58,59,60]), whereas recent computational and process-based approaches substantially improve reliability (r up to 0.64–0.82) and support the task’s usefulness for studying adaptive decision-making over time [61,62].

**Figure 2:**
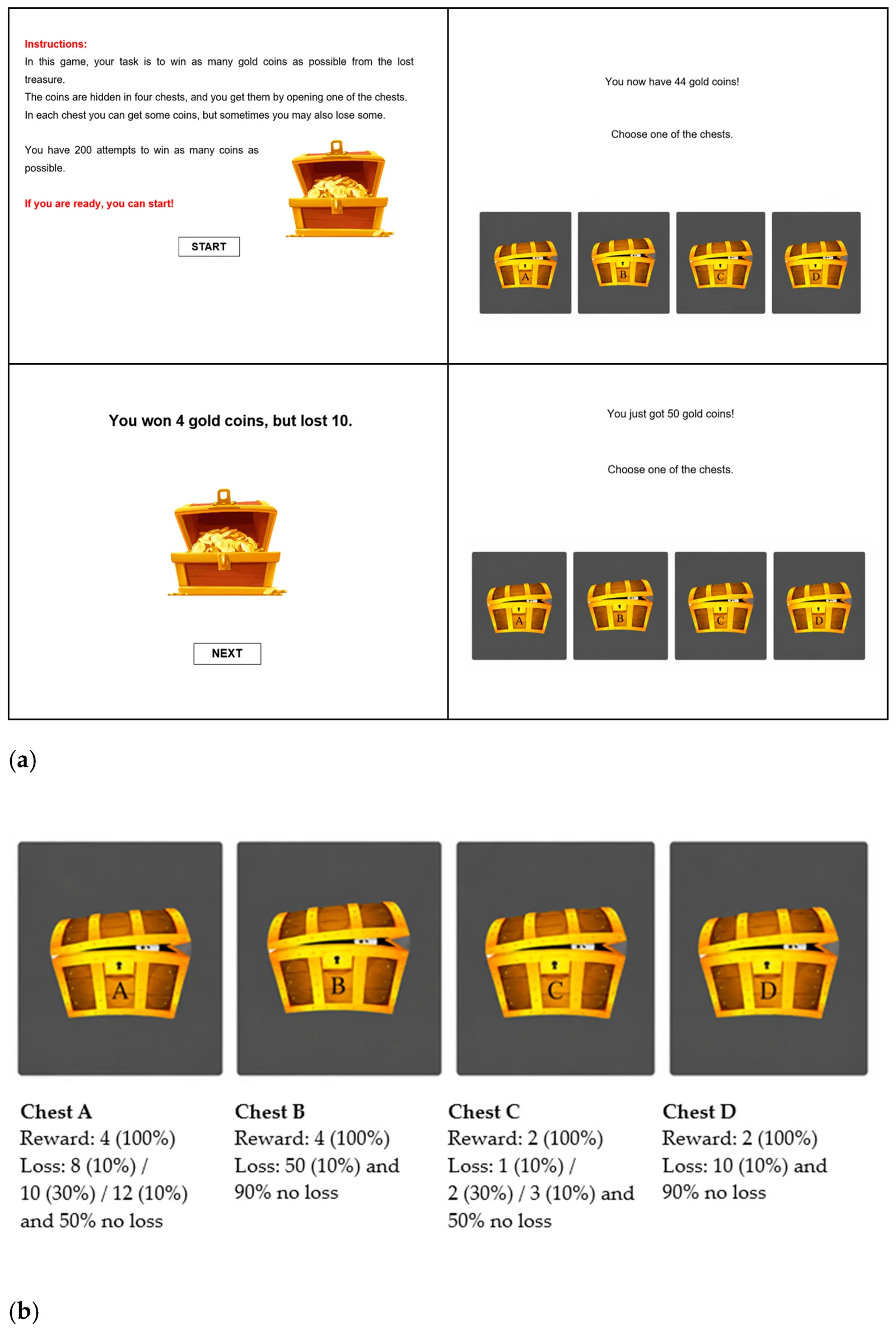
The Iowa Gambling Task—Treasure Hunt version: (**a**) example of visual interaction in an IGT game with chests; (**b**) statistical probabilities of reward and loss for chests A–D.

To assess affective decision-making a child-friendly computerized version of the Iowa Gambling Task (IGT) [63] was used: “Treasure Hunt”. This version involves 200 trials, administered continuously as a single task. For analysis, these trials were divided into five consecutive blocks of 40 trials each. Participants repeatedly chose between four decks (in the form of chests) with varying risk–reward profiles. Two chests (A and B) offer high immediate rewards (four coins) but are disadvantageous due to high long-term losses (on average five coins per trial), while the other two (C and D) offer smaller immediate rewards (two coins) but are advantageous in the long run (average loss is one coin per trial) (Figure 2b).

Children have to collect as many coins as possible, so, in each round, they have to choose one chest, rewarding the child with coin gain/loss. Children received no prior information about the profitability of chests and had to learn through trial and error. Immediate feedback was provided after each selection (Figure 2a). Our relevant indicators are calculated from raw counts of particular chest selections.

Count A (#A), Count B (#B), Count C (#C), and Count D (#D) represent the raw frequency of selections from a particular chest across the entire task (#A + #B + #C + #D = 200). These raw counts serve as the base for derived indices. We calculated two types of indices: outcome-based performance indicators (net score) and process-based indicators, more precisely, feedback-dependent switching indices (e.g., learning index).

*Outcome-based performance indicators*
  - Net score (NS)—operationalized as a cumulative across-task measure of decision-making preference—was calculated as the total difference between the count of advantageous and disadvantageous chest selections over the entire IGT:
    *NS* = (#*C* + #*D*) − (#*A* + #*B*)

A higher NS indicates a predominant preference for long-term advantageous decisions and suggests effective integration of negative feedback. Conversely, a lower or negative NS reflects prioritization of immediate rewards despite long-term losses, which may be associated with reduced feedback sensitivity or underdeveloped delay-of-gratification abilities. Because NS aggregates performance across the entire task, it provides an overall measure of feedback-guided decision-making but does not differentiate between early maladaptive responding and later learning and therefore may obscure within-session shifts in strategy. However, it remains useful for summarizing global decision bias and is frequently used as a primary IGT outcome metric in developmental and clinical studies [56,64].

*Process-based indicators—Feedback-dependent switching indices*

To describe the learning progress across the task, block-wise net scores were computed for each of the five consecutive 40-trial blocks. For each i-th block (out of all five blocks), the net score was calculated as [63]

*NS_i_* = (#*C_i_* + #*D_i_*) − (#*A_i_* + #*B_i_*)

- Learning index (LI) was defined as the difference between the net score in the last block and the net score in the first block:
  *LI* = *NS*_5_ − *NS*_1_

Higher positive values indicate a shift toward more advantageous decision-making over time, negative values indicate a shift toward more disadvantageous decision-making, and values around zero indicate little or no overall change between the beginning and the end of the task. Thus, LI captures the overall direction of learning across the task. Although LI provides a theoretically motivated and parsimonious index of directional learning, it relies on only two data points and may therefore be sensitive to random fluctuations in early- or late-block performance and to regression to the mean. Future research should consider examining the full block-wise learning curve to provide a more complete characterization of feedback-based adaptation across the task.

To examine immediate feedback-related adjustment, each eligible trial-to-trial transition was classified according to the outcome of trial *t* and the participant’s choice on trial *t* + 1. Accordingly, four transition types were distinguished: loss-stay (LSt), loss-shift (LSh), win-stay (WSt), and win-shift (WSh). In the present study, however, the primary analyzed feedback-dependent index was lose-shift, reflecting switching to another chest after a net negative outcome on the preceding trial.

- Lose-shift rate (LSR) was defined as a negative-feedback-dependent switching index. It represents the proportion of eligible trials following a loss on which the child switched to a different chest. In our scoring, loss was operationalized as a net negative outcome on the preceding trial (NetGain < 0); the magnitude of the loss was not taken into account.
  *LSR* = #*LSh*/(#*LSt* + #*LSh*)

Higher LSR values indicate a greater tendency to change choice immediately after negative feedback, and thus greater sensitivity to negative outcomes.

Note: Although LI, LSR, and RLE all capture aspects of feedback-related behavior on the IGT, they reflect distinct processes operating at different temporal scales. LI reflects the overall direction of learning across the task—whether the child’s decision-making shifted toward more advantageous choices from the first to the last block. LSR captures immediate trial-to-trial adjustment following negative feedback—how readily the child switched to a different chest after a loss. RLE reflects general switching behavior between deck categories across all 200 trials, regardless of whether a switch followed a loss or a win. Together, these indices provide complementary perspectives on how children process and respond to feedback during risky decision-making. This task therefore measures components of Hot EFs such as reward/punishment sensitivity and feedback learning [37,65].

- Reversal learning effect (RLE)—ability to abandon an unprofitable strategy based on feedback [66] and represent the shift in the prepotent response during the learning process [67]. RLE was defined as the degree to which child shifted from initially disadvantageous choices in decks A and B toward advantageous choices in decks C and D. Higher values indicate a stronger adaptation of decision-making behavior based on accumulated feedback and a greater ability to abandon initially attractive but disadvantageous response patterns in favor of long-term beneficial choices. This index reflects feedback-based learning and behavioral adaptation under uncertainty.

### 2.3. Statistical Analysis

Hypotheses H1 and H2 were tested using theory-driven hierarchical linear regression models with forced entry (ENTER). In all models, age was entered in Step 1 as a covariate in order to account for shared developmental variance in both Cool and Hot executive processes. Although sex was considered as an additional covariate, it was not included in the final models, as no significant sex differences were observed in either WCST or IGT performance in the present sample. In Step 2, WCST predictors specified by the relevant hypothesis were entered simultaneously. The primary evidence for each hypothesis was the incremental variance explained by WCST predictors beyond age, quantified by ΔR^2^ and evaluated using the F-change test. Each IGT outcome was analyzed in a separate model (for overview see Table 1).

For H1, Step 2 included WCST indices intended to capture cognitive flexibility and set maintenance: perseverative errors (PEs), non-perseverative errors (NPEs), and conceptual level responses (CLRs). Failure to maintain set (FMS) was examined at the bivariate level but was not included as a predictor in the regression models. We argue that FMS differs conceptually from the primary flexibility indices: whereas PE, NPE, and CLR reflect the process of identifying and adapting to new rules, FMS captures inconsistency in applying a rule that has already been established. Given that H1 concerned rule-learning flexibility rather than set maintenance per se, FMS was retained as a descriptive bivariate index only.

Failure to maintain set (FMS) was examined at the bivariate level but was not included as a predictor in the regression models, as the primary focus of H1 was on indices directly reflecting flexible rule application.

These models were fitted to net score (NS) as the primary indicator of overall adaptive decision-making performance.

For H2 (feedback utilization), we prioritized IGT outcomes that are directly driven by feedback-based adjustment and learning over time. Specifically, we modeled (1) the lose-shift rate (LSR) as a negative-feedback-dependent switching index and (2) the learning index (LI) as a summary measure of change in advantageous decision-making between the beginning to the end of the task. For H2, Step 2 included perseverative errors (PEs), conceptual level responses (CLRs), and post-loss reaction time (plRT) as WCST indices reflecting feedback sensitivity, rule conceptualization, and behavioral adaptation following negative outcomes. Because some IGT outcomes required specific trial sequences or complete block-wise data, sample size varied slightly across models when an index could not be computed for all participants. Therefore, each model was estimated using an available-case approach for the relevant outcome, and the model-specific N was reported where appropriate.

Model diagnostics included checks for multicollinearity (VIF/tolerance), influential cases (Cook’s distance; using the 4/N heuristic as a screening rule), and residual-level diagnostics (including Durbin–Watson where reported). Where diagnostics suggested potentially influential observations, results were considered for sensitivity analysis. Given the modest sample size and the estimation of multiple models, statistically significant findings were interpreted with particular attention to effect size, incremental variance, and overall model fit rather than to isolated coefficients alone.

### 2.4. Procedure

Data were collected using the REDCap© electronic tool (v2022–v2024), operated by Comenius University in Bratislava [68] and GORILLA Experiment Builder software© (v2022–v2024), from February 2022 to September 2024 as part of a broader research project.

Participants were recruited from local schools and were tested individually in person. Children were tested digitally via a tablet. Instructions for the games (IGT and WCST) were given in writing and verbally. Before the game, a description of the game instructions was displayed on the tablet. An administrator was always present to make sure that the children understood the instructions, and if necessary, they were retold or explained. Both games were played in one session with an average duration of 30 min.

### 2.5. Ethical Aspects

This study was approved by the Ethics Committee of the Faculty of Arts, Comenius University in Bratislava (decision no. EK/07/2021) as part of a research project. Ethical standards were followed, including informed parental consent, child agreement, and minimal distress.

## 3. Results

Prior to regression analyses, zero-order correlations between all relevant WCST and IGT variables were examined to evaluate the plausibility of hypothesized relationships at the bivariate level. Age was included to assess its associations with both sets of variables. The full correlation matrix for variables included in the hypotheses is presented in Table 2.

Age showed significant positive associations with conceptual level responses (CLRs) (r = 0.358, 95% CI [0.163, 0.526], *p* < 0.001) and significant negative associations with perseverative errors (PEs) (r = −0.264, 95% CI [−0.447, −0.060], *p* = 0.012) and non-perseverative errors (NPEs) (r = −0.390, 95% CI [−0.553, −0.199], *p* < 0.001), confirming expected developmental improvements in WCST performance with age. Age did not show significant associations with failure to maintain set (FMS) (r = −0.047, 95% CI [−0.252, 0.161], *p* = 0.657). Age did not show significant bivariate associations with any IGT outcome (all r ≤ 0.192, all *p* ≥ 0.070), suggesting that, within this sample, age-related variance in Hot EFs performance was limited.

Among WCST predictors, post-loss reaction time (plRT) was the only variable showing significant bivariate associations with IGT outcomes—specifically with net score (NS) (r = −0.274, 95% CI [−0.459, −0.068], *p* = 0.010) and learning index (LI) (r = −0.300, 95% CI [−0.481, −0.094], *p* = 0.005). The core WCST flexibility indices—perseverative errors (PEs), non-perseverative errors (NPEs), and conceptual level responses (CLR)—showed negligible and non-significant associations with all IGT outcomes (all r ≤ 0.187, all *p* ≥ 0.078). Failure to maintain set (FMS) showed significant positive bivariate associations with reversal learning effect (RLE) (r = 0.245, 95% CI [0.031, 0.437], *p* = 0.026), learning index (r = 0.248, 95% CI [0.042, 0.434], *p* = 0.019), and loss-shift rate (LSR) (r = 0.219, 95% CI [0.004, 0.415], *p* = 0.046), but was not entered into regression models, as it falls outside the primary predictive framework of H1 and H2.

This pattern suggests that, at the zero-order level, IGT performance is largely unrelated to WCST-based measures of cognitive flexibility, with the exception of plRT and—at the bivariate level only—FMS.

### Regression Analyses

Given the absence of bivariate associations for most WCST flexibility indices, hierarchical regression analyses were conducted selectively, focusing on outcomes and predictors where both theoretical rationale and bivariate evidence were sufficient. Hierarchical regression models were specified prior to data analysis based on theoretical grounds. Predictor sets for H1 and H2 were defined a priori according to the theoretical distinction between cognitive flexibility indices and feedback-sensitivity indices. Bivariate correlations were reported as preliminary descriptive analyses to contextualize the plausibility of hypothesized relationships, and did not determine predictor selection. Given the theory-driven, confirmatory structure of the analyses—two a priori specified hypotheses tested using hierarchical regression—corrections for multiple comparisons were not applied, consistent with standard practice in confirmatory regression research. Effect sizes (β, R^2^, ΔR^2^) and 95% confidence intervals are reported throughout to facilitate independent evaluation of the practical significance of findings. The sole statistically significant finding (plRT → LI, ΔR^2^ = 0.102, *p* = 0.030) should nonetheless be treated as preliminary, pending replication.

In all models, age was entered in Step 1 as a covariate, and WCST predictors were entered simultaneously in Step 2. Although sex was considered as an additional covariate, it was not included in the final models, as no significant sex differences were observed in either WCST or IGT performance in the sample. The primary criterion for evaluating each hypothesis was the incremental variance explained by WCST predictors beyond age (ΔR^2^).

**H1.**  *WCST flexibility indices and global IGT performance.*

To test H1, perseverative errors (PE), non-perseverative errors (NPE), and conceptual level responses (CLR) were entered as Step 2 predictors of IGT net score (NS)—the primary indicator of overall decision-making preference across the task.

Age alone did not significantly predict net score (R^2^ = 0.037, F(1, 88) = 3.37, *p* = 0.070). The addition of WCST flexibility indices in Step 2 did not produce a statistically significant improvement in model fit (ΔR^2^ = 0.031, F(3, 85) = 0.933, *p* = 0.428), and the full model was not significant (R^2^ = 0.068, adjusted R^2^ = 0.024, F(4, 85) = 1.54, *p* = 0.198). None of the WCST predictors showed a significant unique association with net score (NS) (PE: B = 1.855, 95% CI [−0.866, 4.576], *p* = 0.179; NPE: B = −0.244, 95% CI [−2.385, 1.897], *p* = 0.821; CLR: B = 0.690, 95% CI [−1.422, 2.802], *p* = 0.518). Multicollinearity was acceptable (VIFs = 1.00–2.28). The results do not support H1. WCST-based flexibility indices did not explain incremental variance in global IGT decision-making performance beyond age, consistent with the absence of bivariate associations observed in the preliminary analyses.

**H2.**  *WCST feedback-related indices and IGT learning.*

H2 predicted that WCST feedback-related indices—perseverative errors (PEs) and post-loss reaction time (plRT)—would explain incremental variance in IGT outcomes reflecting feedback-based learning and adjustment (LI, LSR).

IGT Learning Index (LI)

Age alone did not significantly predict LI (R^2^ = 0.017, F(1, 84) = 1.44, *p* = 0.233). Adding WCST feedback-related predictors in Step 2 significantly improved model fit (ΔR^2^ = 0.102, F(3, 81) = 3.14, *p* = 0.030), yielding a significant full model (R^2^ = 0.119, adjusted R^2^ = 0.076, F(4, 81) = 2.74, *p* = 0.034). Within the full model, post-loss reaction time (plRT) was the only significant unique predictor (B = −0.009, 95% CI [−0.015, −0.003], *p* = 0.003, β = −0.322), indicating that slower post-error responding on the WCST was associated with a smaller increase in net score (NS) from the first to the last IGT block—that is, with a less pronounced shift toward advantageous choices over the course of the task. Age (B = −8.862, 95% CI [−19.845, 2.121], *p* = 0.112), PE (B = 0.435, 95% CI [−0.519, 1.390], *p* = 0.367), and CLR (B = 0.154, 95% CI [−0.507, 0.815], *p* = 0.644) were not significant unique predictors. Multicollinearity was low (VIFs = 1.03–1.50).

Post-loss reaction time (plRT) on the WCST, as an index of behavioral sensitivity to negative feedback in a Cool EF context, uniquely predicted the degree of learning-related improvement in Hot EF performance beyond age.

IGT Loss-Shift Rate (LSR)

For LSR, the age-only model was not significant (R^2^ = 0.000, F(1, 81) = 0.025, *p* = 0.875). Adding WCST feedback-related predictors did not significantly improve model fit (ΔR^2^ = 0.003, F(3, 78) = 0.072, *p* = 0.975), and the full model was not significant (R^2^ = 0.003, adjusted R^2^ = −0.048, F(4, 78) = 0.060, *p* = 0.993). No predictors showed significant unique associations (all *p* > 0.81). Multicollinearity was low (VIFs = 1.00–1.56).

These results do not support H2 for immediate trial-to-trial feedback adjustment. Unlike LI, which captures gradual integration of feedback across the entire task, LSR reflects reactive switching after individual losses—a more immediate process that appears to operate independently of Cool EF feedback sensitivity as measured by the WCST.

Taken together, the pattern of results suggests that WCST-based measures of cognitive flexibility—perseverative errors (PEs), non-perseverative errors (NPEs), and conceptual level responses (CLRs)—were unrelated to IGT performance at both the bivariate and multivariate level, providing no support for H1. Partial support was found for H2: post-loss reaction time (plRT) on the WCST uniquely predicted the IGT learning index (LI) beyond age (ΔR^2^ = 0.102, *p* = 0.030). This indicates that greater sensitivity to negative feedback consequences—reflected in post-error slowing on the Cool EF task—is associated with a less pronounced shift toward advantageous decision-making over the course of the IGT, a Hot EF task.

## 4. Discussion

The aim of this study was to examine the relationship between selected Hot and Cool EFs. The Wisconsin Card Sorting Test (WCST) captured Cool EF—cognitive flexibility (and its partial variables such as PE, NPE, plRT)––as well as the child’s ability to maintain a learned rule (CLR, FMS examined descriptively at the bivariate level). Using the Iowa Gambling Task (IGT), we measured Hot EF—decision-making in uncertain situations (represented by the variables NS, LSR, and LI). The focus of the research was on cognitive flexibility and the use of feedback from the WCST and their impact on IGT results specifically: adaptive IGT performance (H1), feedback-based adjustment, and learning over time (H2).

The present findings provide limited support for the formulated hypotheses. Therefore, they also provide weak evidence for the idea that there is a broad behavioral overlap between WCST and IGT performance in middle childhood. After accounting for age, most WCST indices did not explain additional variance in the IGT outcomes, suggesting that the relationship between cognitively mediated flexibility and affectively charged decision-making is weak at the level of global behavioral performance. Rather than supporting a general link between Cool and Hot executive functioning, the results indicate a more selective and outcome-specific pattern of association.

Despite expectations, hypothesis H1 was not supported. WCST measures of cognitive flexibility (CF) and set maintenance––perseverative errors (PEs), non-perseverative errors (NPEs), conceptual level responses (CLRs)––did not predict overall IGT performance (net Score—NS) across age. These findings suggest that traditional WCST measures of Cool EFs may not be directly related to adaptive decision-making in situations characterized by uncertainty (resp. reward and punishment).

At the bivariate level, FMS showed significant positive associations with LI (r = 0.248), LSR (r = 0.219), and the reversal learning effect (RLE) (r = 0.245), suggesting that children who had more difficulty maintaining a correct set on the WCST tended to show more feedback-reactive behavior on the IGT. However, since FMS the associations are modest, this pattern warrants cautious interpretation.

Within the H2 hypothesis, one of the interesting results is indicative, concerning the loss-shift rate (LSR). In the model, neither age nor WCST feedback-related indices were significant predictors. This result can also be interpreted as the tendency of children to change their choices in the IGT depending on previous results and may be shaped to a greater extent by general developmental factors than by specific abilities related to, e.g., feedback processing (which are captured by the WCST). This reasoning is also supported by the developmental perspective, because children undergo significant changes in behavioral regulation, frustration tolerance, and other EF-related abilities such as the ability to delay gratification precisely in the investigated age range of 6 to 12 years [54,69,70]. Some of the variability in choice in the IGT may thus naturally reflect broader maturational changes in decision-making regulation rather than the transfer of WCST-type rule-shifting. In this sense, the result suggests that some aspects of performance in the IGT may simply be developmentally general, rather than specifically “WCST-like”.

The clearest overlap was seen in the learning index (LI) in IGT. In this case, the WCST scores explained significant additional variability beyond age. This effect was mediated by post-loss reaction time (plRT) in the WCST, specifically by a negative relationship, i.e., children who responded more slowly after a loss (RT ↑) on the WCST showed less improvement in decision-making quality between the first and the last IGT block (LI ↓); conversely, children who responded faster after negative feedback (RT ↓) showed greater improvement in advantageous choice over the course of the task (LI ↑).

However, the direction of the association is also important to consider. Slower response after negative feedback can be interpreted as a manifestation of increased cognitive control, greater deliberation, or more careful monitoring of performance. At the same time, post-feedback slowing is not a process-specific indicator and may also reflect increased uncertainty, attentional orienting to an unexpected outcome, or less efficient processing of negative feedback. Thus, longer reaction time alone does not allow us to distinguish between adaptive reflection and processing difficulty. In our data, longer post-loss latencies in the WCST were associated with weaker improvement in LI. This pattern suggests that, in the present sample, post-loss slowing was not functionally linked to more adaptive feedback integration in a Hot EF context. This finding does not determine which of these mechanisms underlies the longer reaction time, but it indicates that the functional outcome of post-loss slowing—at least as captured by LI—was less adaptive decision-making rather than more. What may be decisive is whether the processing of feedback leads to productive behavioral reorganization—a question that reaction time data alone cannot resolve.

In specific cases, this may mean that, in some children, a prolonged reaction after negative feedback reflects difficulty translating negative-feedback processing into effective strategic updating or higher uncertainty. Although these children will experience a negative outcome, subsequent processing may be less efficient or less successfully reflected in the improvement of subsequent choices. This supports the view that behavioral self-regulation in effective feedback processing includes not only inhibition, but also the ability to respond flexibly and promptly to changing environmental conditions [71,72,73,74]. This pattern of behavior could also help explain why broader measures of flexibility in the WCST were generally unrelated to performance on the IGT. Traditional WCST scores, such as perseveration errors (PEs), non-perseveration errors (NPEs), and conceptual level responses (CLRs), may capture relatively general aspects of rule-governed flexibility, but may be too distant from the more process-sensitive mechanisms that are relevant to learning under conditions of uncertainty in the IGT.

Conversely, post-loss reaction time (plRT) may capture a narrower adaptation mechanism that is more closely related to how children process negative outcomes across tasks. A similar interpretive caution has been raised regarding reaction-time-based indices in other developmental learning paradigms: in a study of observational learning in early primary school, slower choice-related reaction times were associated with fewer errors during an initial detection phase, yet with more repetitions needed to reach error-free performance during a later exercise phase, suggesting that the functional meaning of response speed depends on the specific process being measured rather than reflecting a single global characteristic such as general efficiency or impulsivity [75]. This process-specific pattern is consistent with the broader developmental literature on attention, where discrete components—such as focused and divided attention—rather than attention as a unitary construct differentially predict children’s learning outcomes and are themselves conditioned by developmental and contextual factors [75,76].

The role of age in the analyzed models was relatively limited. Neither the H1 nor the H2 model yielded a significant contribution of age beyond what was already accounted for at the bivariate level. This suggests that, within the observed age range of 6 to 12 years, developmental factors do not uniformly explain individual differences in IGT performance once task-specific WCST indices are considered.

Several factors may help explain this pattern of weak and selective associations. (1). First, the WCST and IGT differ significantly in task structure. The WCST uses explicit feedback in a relatively structured rule-learning context, whereas the IGT requires gradual learning from probabilistic gains and losses in an affectively more salient context. (2). At the same time, the age range of the sample studied was wide, and developmental changes likely contribute significantly to performance on both tasks. (3). In the context of the “(im)purity” of capturing the variables under investigation, we can assume that some IGT indices, especially those based on switching behavior, may reflect multiple processes simultaneously, including impulsivity, reactivity, uncertainty, and adaptive exploration. Similarly, both tasks are inherently “impure” in nature, with performance in them not only dependent on flexibility or feedback use, but also on attention, inhibition, motivation, response consistency, and sensitivity to reward and loss. This may contribute to the low overlap between the tasks in measured EF dimensions.

Notably, a weak behavioral correlation between two tasks does not necessarily indicate that the underlying processes are independent: robust, well-established cognitive tasks frequently yield low correlations with other measures, not because the underlying constructs are unrelated, but because low between-subject variability limits the reliability needed to detect true associations [77]. Modest convergence between different measures of Hot and Cool executive functioning has similarly been interpreted as reflecting distinct but complementary dimensions of executive functioning, rather than evidence of unrelated systems [78].

In general, the relationship between WCST and IGT in childhood is best understood as weak, age-heterogeneous, and process-specific—a pattern more consistent with gradual integration of cognitive and emotional regulation processes throughout childhood [28] than with either full unity or full independence of Hot and Cool EFs. The most pronounced overlap is in the relationship between post-loss adjustment in WCST and improvement in advantageous decision-making across the IGT as captured by the learning index (LI), highlighting the value of examining how individuals respond to feedback in real time rather than relying solely on global outcome measures.

### 4.1. Results from a Broader Perspective of Previous Research on Unity vs. Diversity

Our findings align partially with existing literature, although there are a limited number of studies directly exploring relationships of Hot and Cool EFs.

Our results are supported by some previous studies. Already, Bachara [79] reported little relationship between the IGT and WCST performance measures. In the study by Overman et al. [80], performance on the WCST in their sample of adolescents was not significantly related to performance on the IGT. Even in their later study [81], a significant correlation between measures of the IGT and performance on WCST was not supported. These findings suggest that cognitive flexibility (CF) and the ability to adapt to new categories (measured by the WCST) may not automatically imply performance in decision-making tasks with risk and uncertainty, such as the IGT. This reasoning is extended by Stocco et al. [82], who propose that decision-making in the IGT is the result of the interaction of two separable cognitive processes: one “central” cognitively demanding process that monitors long-term returns, and the other “lower-level” automatic and fast process sensitive to the frequency and magnitude of losses. According to this model, performance in the IGT can be influenced by other variables (emotional signals, sensitivity to reward/punishment). Similarly, a clinical study by Kodaira et al. [83] reported that children with OCD had significantly worse IGT performance, while their WCST performance was not significantly different compared to the control group. This suggests that decision-making disorders (IGT) may manifest themselves independently of the deficit in EFs measured by the WCST.

However, the results from previous research are not unambiguous. As we have already mentioned, a considerable number of studies report that EF components measured by the WCST can predict parts of IGT performance [44,45,46]. Dong, Du, and Qi [37] used ERP (event-related potential) measurements to demonstrate that WCST performance was able to predict selected aspects of IGT performance; moreover, they claim to recognize the cognitive component of the WCST that effects IGT. Also, children with enhanced cognitive flexibility achieve better overall IGT scores and are prone to choosing more advantageous cards [84]. The learning index (LI) also has been linked to higher cognitive flexibility in performance tasks [37], which is partially consistent with our finding that plRT on the WCST predicted LI on the IGT—though the mechanism appears to be process-specific rather than reflecting a broad Cool–Hot EF overlap.

Games like the IGT and the WCST may require a combination of cognitive processes [38]. In addition, performing the IGT task involves complex interdependent cognitive, response, and motivational processes [85]. To determine which processes underpin individual learning and behavior in these tasks, some researchers advocate a cognitive modelling approach [86].

The fact that the study was conducted with children aged 6–12 may also play a significant role. In children, EFs are still developing, and the developmental trajectories of Hot and Cool EFs may accelerate differently [87,88,89,90]. At certain developmental stages, these systems may function relatively independently of one another. This may suggest that emotional decision-making may be less connected to cognitive flexibility at some points in development than at others (earlier or even later in life).

However, the strength of task-specific performance indicators varies and is not consistent. This inconsistency is also highlighted in work by Moriguchi and Phillips [24]. They also suggest the necessity of further research to clarify whether such variability stems from methodological limitations, specific EF components, or broader conceptual issues within the Hot/Cool EF framework. Welsh and Peterson [90] suggest similar objections. Moreover, the division of EFs itself is more didactical than practical, and dimensionality suggests that differences in EF components are more continual than categorical.

Overall, these findings support a partial integration model of EFs in middle childhood, in which the Cool and Hot EF dimensions interact dynamically rather than operate in isolation.

### 4.2. Limitations

The cross-sectional research design we used does not allow us to directly observe developmental trajectories or causal relationships, nor to determine the “order” of changes in performance that are related to performance on the WCST and the IGT. Although age was included as a covariate, this cannot fully capture developmental dynamics that may be nonlinear and may differ between individual components of cognitive flexibility, feedback processing, etc. This is particularly relevant given the relatively wide age range (6 to 12 years) of the sample, which likely includes children at different stages of self-regulatory and decision-making maturation [72]. Although age was entered as a linear covariate in the primary models as pre-specified, a supplementary robustness analysis revealed a significant non-linear age effect on LI (quadratic term: B = −2.46, *p* = 0.049, ΔR^2^ = 0.042), suggesting an inverted-U developmental pattern in feedback-based learning across the 6–12 year range, with children in the middle of the age range showing greater improvement in advantageous decision-making across IGT blocks than younger or older children. No significant quadratic age effect was found for NS (*p* = 0.622). Importantly, the plRT effect remained virtually unchanged under the quadratic specification (B = −0.009, *p* = 0.004, β = −0.305 vs. B = −0.009, *p* = 0.003, β = −0.322 in the primary model), confirming that the primary H2 finding is robust to the specification of the age covariate. The non-linear developmental pattern observed for LI warrants further examination in future studies with larger, age-stratified samples.

While the sample size was sufficient for detecting moderate effects in the primary models, it limits the feasibility of detailed age-stratified analyses; potential age-related differences in Hot and Cool EF functioning could therefore not be fully explored, and findings should be considered exploratory.

Although we tested specific hypotheses, the limited existing literature on this topic and our sample size mean these results are best viewed as preliminary.

The nature of the tasks used played an equally important role. Both tasks were administered in computerized, developmentally adapted versions, which increases their suitability for children, but may also limit direct comparability with the results of studies using standard versions of the WCST and the IGT. This is especially true for the children’s version of the IGT, in which motivational and behavioral characteristics of performance may be partially influenced by the game setting of the task. Furthermore, neither task used represents a pure measure of a single psychological construct, as we have already described above.

Task impurity thus poses a substantive constraint on construct-level interpretation of the present findings. The WCST draws on working memory, inhibition, and attentional control in addition to cognitive flexibility, while the IGT engages motivation, reward sensitivity, and emotional processing alongside feedback-based learning. As a result, a weak or absent WCST–IGT association does not provide a straightforward indication that Cool and Hot EFs are separable constructs—it may equally reflect that task-specific variance in each instrument attenuated the cross-task signal. Conversely, the plRT–LI association, while modest, cannot be attributed solely to shared executive functioning—it may partly reflect shared variance in general processing speed or response consistency across tasks. Disentangling these sources of variance would require designs using multiple task-based indicators of each EF construct, allowing shared executive variance to be separated from task-specific method variance. No formal reliability data are available for the derived indices in the present study. This is a known limitation of process-based IGT indices more broadly—test-retest reliability tends to be modest, particularly in child and adolescent samples, where decision-making is inherently less stable. The stability of plRT and LI in the present sample is therefore unknown, and this uncertainty should be considered when interpreting the plRT–LI association.

The implementation of testing in a single session can also be considered a limit. Although fatigue was monitored using a non-verbal self-report scale, the influence of momentary motivation, attentional fluctuations, habituation to the test situation, or order effects between task cannot be completely ruled out. In children, such factors may affect performance more significantly than in adults—even if the order was randomized.

### 4.3. Recommendations for Future Research and Practice

Future research should focus on more detailed examination of process indicators of executive functioning, such as reaction time dynamics or behavioral analysis at the trial level: such process-based measures may provide insight into underlying decision-making patterns not captured by outcome-based scores alone [91], may generalize more consistently across decision-making tasks [92], and are often neglected when analyses rely solely on global performance scores [38]. It would also be appropriate to combine different methodological approaches, including behavioral tasks and rating scales, which could contribute to a more comprehensive understanding of executive processes. Our findings suggest that broad global indicators of performance may mask important differences in how children process and use feedback; therefore, future research should distinguish more clearly between overall task performance and more immediate, process-sensitive indicators of adaptation.

For future research, we would also recommend trying to verify the results with other tasks/instruments that measure the same broader constructs of cognitive flexibility and decision-making in uncertain situations. In the case of decision-making in uncertain situations, tasks such as the Cups Task [93,94] and the Balloon Analogue Risk Task for children (BART-C) may be appropriate [95]. For cognitive flexibility, we would recommend tasks such as the Dimensional Change Card Sort (DCCS) [96], the Flexible Item Selection Task (FIST) [97], the NEPSY-II “Switching” subtest [98], the PC game IED (Intra/Extra-Dimensional Set Shift) [99], or the new Executive Brain Battery (EBB) measuring Hot and Cool EFs in middle childhood [54].

Future studies should also aim to separate sensitivity to negative versus positive feedback more explicitly, as these processes may contribute differently to adaptive decision-making.

It could be interesting to expand the age range of the probands, so that differences in preschool age, in late adolescence, adulthood, and older age could be examined separately.

Future research would also benefit from longitudinal designs that would make it possible to examine whether the relationship between Hot and Cool executive functioning changes across development. Such designs could help clarify whether the weak overlap observed in the present study reflects a temporary developmental dissociation, age-related heterogeneity, or limited shared variance between the constructs.

Taken together, our findings show that adaptive decision-making is less dependent on traditional measures of cognitive flexibility and more related to effective processing of negative feedback. These findings highlight the importance of dynamic aspects (process-based indicators) of executive functioning and may have implications for the design of interventions aimed at developing executive and self-regulatory skills in children.

## 5. Conclusions

The findings suggest that traditional WCST indices of cognitive flexibility do not predict IGT performance; however, process-level sensitivity to negative feedback (as reflected by post-loss reaction time—plRT) may play a more relevant role in adaptive learning during decision-making in uncertain situations. It can be argued that the association between the WCST and the IGT during childhood is best described as weak, subject to developmental changes, and specific to the process. The plRT–LI association, which constitutes the study’s primary significant finding, should be treated as preliminary, pending replication in larger, pre-registered studies.

## Figures and Tables

**Table 1:** Overview of hierarchical regression models by hypothesis: outcomes, step 2 predictors, and analytic sample sizes.

Hypothesis	Outcome	Step 2 Predictors	Analytic N
H1	Net score (NS)	PE, NPE, CLR	N = 90
H2	Learning outcome/LI	PE, CLR, plRT	N = 86
H2	Loss-shift rate (LSR)	PE, CLR, plRT	N = 83

Note. The stated overall N = 90 reflects children meeting all inclusion criteria who completed both tasks. Model-specific N varies due to availability of derived indices under the available-case approach.

**Table 2:** Zero-order correlations between age and relevant WCST and IGT variables.

	NS	LI	LSR	RLE
Age	−0.192	0.127	0.018	0.178
	[−0.384, 0.016]	[−0.084, 0.327]	[−0.199, 0.232]	[−0.039, 0.379]
PE	0.187	0.021	−0.007	0.003
	[−0.021, 0.379]	[−0.188, 0.228]	[−0.222, 0.209]	[−0.213, 0.218]
NPE	−0.017	−0.012	−0.048	0.056
	[−0.223, 0.191]	[−0.219, 0.197]	[−0.261, 0.170]	[−0.162, 0.269]
CLR	−0.049	0.040	0.012	−0.114
	[−0.254, 0.160]	[−0.169, 0.246]	[−0.204, 0.227]	[−0.321, 0.105]
plRT	−0.274 *	−0.300 **	0.001	−0.091
	[−0.459, −0.068]	[−0.481, −0.094]	[−0.219, 0.220]	[−0.305, 0.131]
FMS	0.136	0.248 *	0.219 *	0.245 *
	[−0.073, 0.334]	[0.042, 0.434]	[0.004, 0.415]	[0.031, 0.437]

NS = net score; LI = learning index; LSR = loss-shift rate; RLE = reversal learning effect; PE = perseverative errors; NPE = non-perseverative errors; CLR = conceptual level responses; plRT = post-loss reaction time on WCST; FMS = failure to maintain set. * *p* < 0.05. ** *p* < 0.01.

## Data Availability

The data presented in this study are available on request from the corresponding author. (The data are not publicly available, due to privacy restrictions).

## Abbreviations

The following abbreviations are used in this manuscript:

Abbreviation	Meaning
ASD	Autism spectrum disorder
BART-C	Balloon Analogue Risk Task for Children
CF	Cognitive flexibility
CLR	Conceptual level responses (WCST)
DCCS	Dimensional Change Card Sort
dlPFC	Dorsolateral prefrontal cortex
EBB	Executive brain battery
EFs	Executive functions
FIST	Flexible item selection task
FMS	Failure to maintain set (WCST)
IED	Intra/Extra-Dimensional Set Shift
IGT	Iowa Gambling Task
LI	Learning index (IGT)
LSR	Lose-shift rate (IGT)
NPE	Non-perseverative error (WCST)
NS	Net score (IGT)
PE	Perseverative error (WCST)
plRT	Post-loss reaction time (WCST)
RLE	Reversal learning effect (IGT)
SLD	Specific learning disorder
WCST	Wisconsin Card Sorting Test
WM	Working memory
